# Model Selection and the Molecular Clock

**DOI:** 10.1371/journal.pbio.0040151

**Published:** 2006-05-16

**Authors:** Oliver G Pybus

## Abstract

A brief overview of the methods used to determine phylogenetic distances sets the stage for understanding new research published in *PLoS Biology*.

There are no mathematical equations in *On the Origin of Species*. A good thing too, you might think, and it is undoubtedly true that Darwin's clear and flowing narrative style helped ensure the popularity of his writings. Modern research in evolutionary biology can make for less easy reading. Much of it concerns the development of an expanding arsenal of mathematical and statistical techniques, necessary to do battle with the relentless onslaught of gene and genome sequences. Of course, the discrete, ordered nature of genetic information and the stochastic character of Mendelian inheritance have naturally lent themselves to numerical analysis. Consequently, the mathematical foundations of evolutionary genetics have, somewhat unusually for biology, tended to precede the data to which they are applied. *The Genetical Theory of Natural Selection* by R. A. Fisher, published only fifty years after Darwin's death, is full of equations [ 1].

The simplest weapon in the armoury of evolutionary genetics is genetic distance, a measure of the number of evolutionary changes between sequences from different organisms. Genetic distances can be calculated for a pair of sequences by simply counting the number of nucleotides or amino acids that differ between them. Unfortunately, this approach underestimates the amount of evolutionary change because it does not account for the fact that each site may change more than once during evolutionary history. Statistical tools, called nucleotide or amino acid substitution models, are therefore used to estimate genetic distances between sequences. There is a bewildering hierarchy of substitution models available, each making a different and specific set of assumptions about the evolutionary process of sequence change [ 2]. The simplest models assume that all types of mutation are equivalent and that all sites in a sequence change at the same rate. More complex models loosen these assumptions, allowing heterogeneity in the process of sequence change, but they can be reliably applied to larger datasets only. The task of deciding amongst these competing models is known as statistical model selection and can be thought of as a trade-off between model accuracy and model complexity. The degree to which a model fits the data at hand (accuracy) is always improved by adding more parameters (complexity), but since the amount of data remains constant the statistical uncertainty about each parameter increases. In addition, the biological meaning of each parameter becomes harder to decipher so the explanatory power of the model decreases ( Figure 1). Thus the chosen model should have enough parameters to adequately explain the data—but no more. Once an appropriate model is chosen, genetic distances are combined using other statistical techniques to generate a phylogenetic tree of the sequences being studied [ 2]. The lengths of the branches in the phylogeny thus represent estimated numbers of sequence changes ( Figure 2A).

**Figure 1:**
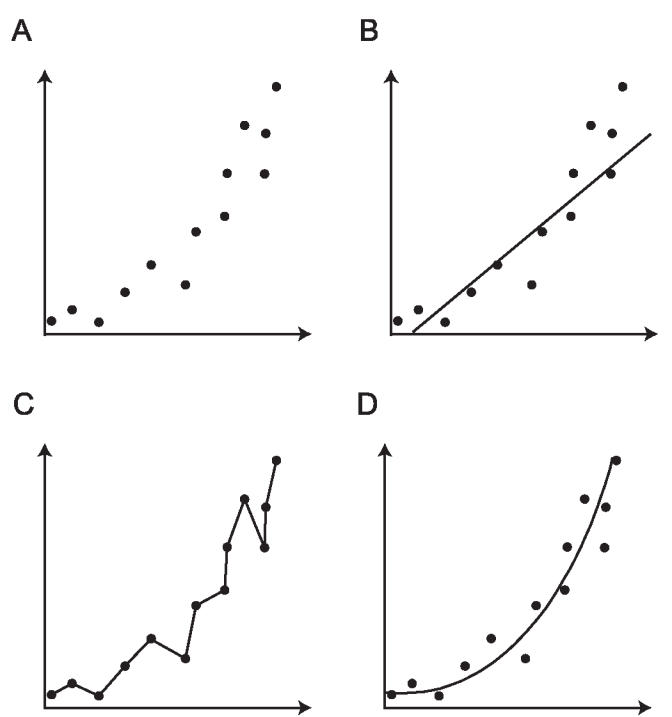
An Illustration of the General Properties of Model Selection (A) A hypothetical dataset consisting of thirteen points plotted on two axes. (B) A simple model, represented by a straight line through the points. This model has few parameters but does not fit the data particularly well. (C) A very complex model, which fits the data almost perfectly but has too many parameters. The estimated parameters tell us little about the biological process that gave rise to the data. (D) A model with an intermediate number of parameters represented by a curve. This fits the data well but still has relatively few parameters and therefore has greater explanatory power.

**Figure 2:**
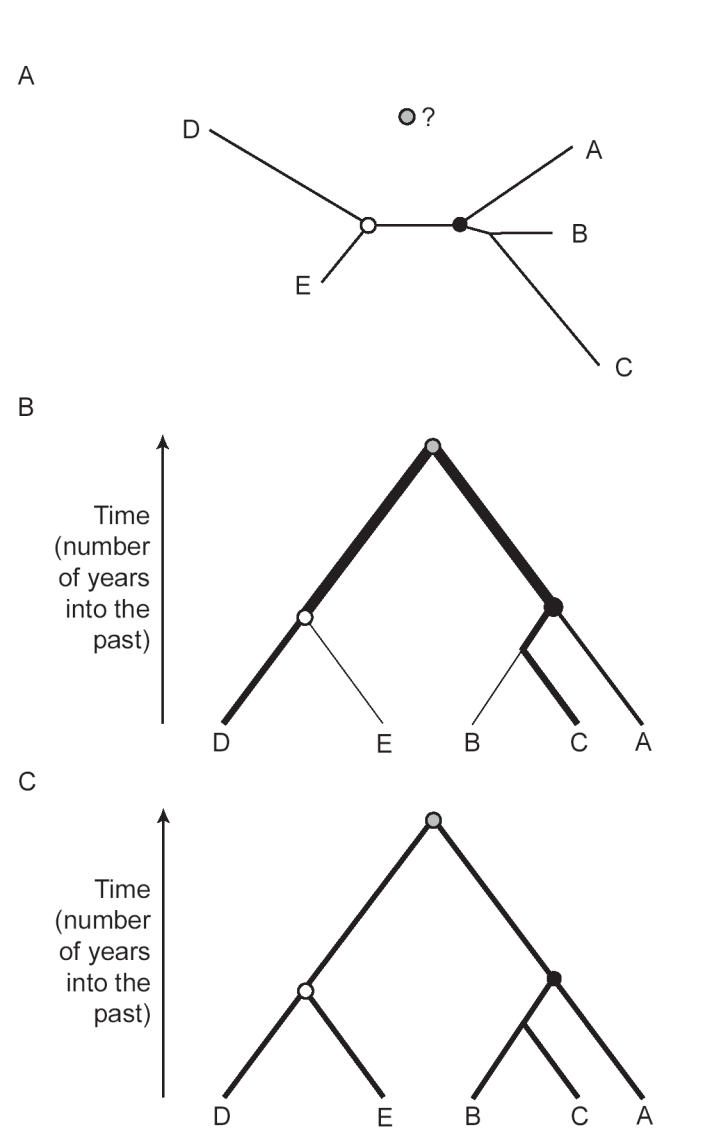
Example Phylogenies, Each Representing the Shared Ancestry of Five Organisms The circles represent common ancestors: the common ancestor of A, B, and C (black circle), the common ancestor of D and E (white circle), and the overall common ancestor of all five (grey circle). (A) A phylogeny generated using the no-clock model of evolution. Such phylogenies are “unrooted”; that is, the position of the overall common ancestor cannot be identified. Branch lengths represent genetic distance, not time. (B) A phylogeny generated using the relaxed-clock model. The overall common ancestor is identified; hence the phylogeny is “rooted”. Branch lengths represent time. The thickness of each branch indicates the rate of evolution of that branch. (C) A phylogeny generated using the strict-clock model. This is the same as the relaxed-clock case, except that the rate of evolution is identical for every branch.

However, genetic distances are rather crude indicators of evolutionary history. A small genetic distance between two sequences may suggest a recent common ancestor, but is also consistent with a slower rate of sequence change and a more ancient common ancestor (i.e., genetic distance = evolutionary rate × time). Genetic distances alone are therefore of little use if, for example, we wish to know the age of the common ancestor of mammals, or the rate at which bacterial antibiotic resistance genes evolve. Such questions can be answered only if independent information about rates or divergence times is found. Often paleontology or biogeography can provide a date for one or more points in a phylogeny, which are then used to “calibrate” the timescale for the rest of the phylogeny [ 3, 4]. Less commonly, sequences sampled at different times can provide an estimated rate of evolution; this requires either a very fast evolutionary rate (e.g., rapidly evolving RNA viruses [ 5]) or widely spaced sampling times (e.g., ancient DNA from sub-fossil samples [ 6]). Whatever the source of the independent information, it is usual to calibrate a phylogeny by assuming that all its branches evolve at the same rate—i.e., there is a constant but stochastic “molecular clock” of sequence change. The concept of the molecular clock originated in the early 1960s and has since been used widely, more as a result of its downright usefulness than its biological accuracy, as it is clear that rates of evolution can and do vary considerably among species [ 4, 7]. Evolutionary rates depend on a combination of factors: generation time, population size, metabolic rate, the efficacy of DNA repair, and the degree to which mutations are beneficial or deleterious, all of which may vary among species. As the geneticist Steve Jones recently remarked, evolutionary biologists seem to use the molecular clock “with our fingers crossed” [ 8].

The article by Alexei Drummond, Andrew Rambaut, and colleagues in this issue of *PLoS Biology* [ 9] gives us reason to uncross our fingers a little. The paper describes a new “relaxed” approach to the estimation of phylogeny divergence times. A relaxed molecular clock is a phylogenetic technique that allows the rate of sequence evolution to vary among groups of organisms, or more generally, among different parts of a phylogeny ( Figure 2B). The use of a single rate across the whole phylogeny is termed a “strict” clock ( Figure 2C). Such methods have developed steadily in the past ten years (e.g., [ 10, 11]) and can now be applied to large datasets due to the continued increase in computer processing speed. A common aspect of previous relaxed-clock approaches is that they considered closely related organisms to have similar rates of evolution. On a phylogeny this means that neighbouring branches have more similar rates than distant branches, a property termed “autocorrelation”. The idea that rates of sequence evolution can be “inherited” in this way played an important role in the history and development of evolutionary theory [ 7, 12], and it is well known that viruses, bacteria, and animals evolve at hugely different rates, but the assumption has never been comprehensively tested. Drummond et al.'s new method allows phylogeny branches to vary in rate, but it does not assume these rates are correlated among adjacent branches ( Figure 2). Thus their relaxed clock is slightly more laid-back than its predecessors, and crucially it can estimate the level of autocorrelation in each dataset. A further advantage of their approach is that it simultaneously estimates both phylogeny shape and rate variation among phylogeny branches, two tasks that previously had to be performed separately.

We should note that Drummond et al's paper emphasises the fact that molecular clocks exist as a family of statistical models, analogous to the hierarchy of substitution models discussed earlier, among which the most appropriate model should be chosen. When constructing a phylogeny, many researchers opt not to “enforce” a molecular clock, perhaps believing that they are avoiding having to make any possibly unrealistic assumptions about evolutionary rates. In truth, this “no-clock” approach is equivalent to using an evolutionary model that assumes no limit to the variation in evolutionary rate among branches. In fact, it has a separate evolutionary rate parameter for each branch in the phylogeny. If, as is often the case, rate variation among organisms is not great, then the no-clock model will have an unnecessarily large number of parameters, leading to an increase in statistical uncertainty and, in some circumstances, poorer estimates of phylogeny shape. Drummond et al. analysed five large datasets, containing sequences from bacteria, yeast, plants, animals, and primates, and found that in every case their relaxed-clock model identified the “true” phylogeny slightly more often than the no-clock model. Importantly, the relaxed-clock estimates were more certain than those of the no-clock model, as expected given the greater number of parameters in the latter model [ 9]. A key area of future research will be to investigate these results using statistical model selection theory.

It is perhaps surprising that gene sequences contain sufficient information to estimate as complex a process as evolutionary rate variation among organisms. But it is well known that if phylogenies are constructed using the no-clock model, then the genetic distances of sequences to a shared common ancestor are unequal ( Figure 2A). Since sequences are sampled at the same time (on an evolutionary scale), the times to the common ancestor will be identical for each; hence the variation in genetic distance directly reflects the variation in evolutionary rate since the common ancestor. This valuable information about the evolutionary process is ignored whenever the no-clock model is used, despite it being used for many years in the relative rates test, a statistical test used to detect evolutionary rate variation [ 7].

It is likely that the widespread adoption of relaxed-clock models in phylogenetics will act as a stepping-stone to even more intricate models of sequence change. Work has already begun on combining evolutionary rate variation among organisms with rate variation among genomic sites, so that particular sets of sites are able to evolve quicker or slower on different sets of branches [ 13]. This could be important if certain parts of a gene are under selection in some species but not others. This complex situation, known as heterotachy, is currently the subject of debate amongst phylogeneticists, as it is unclear whether model-based statistical approaches are better than “model-free” parsimony methods that appear not to make assumptions about the evolutionary process [ 13, 14]. In many ways, this debate echoes the relaxed-clock and no-clock comparison discussed above. It is quite possible that in this case, too, the model-free parsimony methods are making implicit assumptions about the nature of rate variation among sites and lineages, but the underlying process is so complicated that it will take time for these assumptions to be fully understood. The complexity of heterotachy will also require larger datasets than are currently used in phylogenetics. But in the midst of a revolution in high-speed genomics, it is not sequence data we are short of, but tools for statistical analysis—and the equations on which they are based.
